# Drug-Induced Metabolic Acidosis

**DOI:** 10.12688/f1000research.7006.1

**Published:** 2015-12-16

**Authors:** Amy Quynh Trang Pham, Li Hao Richie Xu, Orson W. Moe

**Affiliations:** 1 Charles and Jane Pak Center for Mineral Metabolism and Clinical Research, University of Texas Southwestern Medical Center at Dallas, Dallas, TX, 75390-8885, USA; 2 Departments of Internal Medicine, University of Texas Southwestern Medical Center at Dallas, Dallas, TX, 75390-8885, USA; 3 Department of Physiology, University of Texas Southwestern Medical Center at Dallas, Dallas, TX, 75390-8885, USA; 4 Baylor Family Medicine Residency at Garland, University of Texas Southwestern Medical Center at Dallas, Dallas, TX, 75390-8885, USA

**Keywords:** metabolic, acidosis, drug-induced, MALA

## Abstract

Metabolic acidosis could emerge from diseases disrupting acid-base equilibrium or from drugs that induce similar derangements. Occurrences are usually accompanied by comorbid conditions of drug-induced metabolic acidosis, and clinical outcomes may range from mild to fatal. It is imperative that clinicians not only are fully aware of the list of drugs that may lead to metabolic acidosis but also understand the underlying pathogenic mechanisms. In this review, we categorized drug-induced metabolic acidosis in terms of pathophysiological mechanisms, as well as individual drugs’ characteristics.

## Introduction

Metabolic acidosis is defined as an excessive accumulation of non-volatile acid manifested as a primary reduction in serum bicarbonate concentration in the body associated with low plasma pH. Certain conditions may exist with other acid-base disorders such as metabolic alkalosis and respiratory acidosis/alkalosis ^1^.

**Figure 1.**
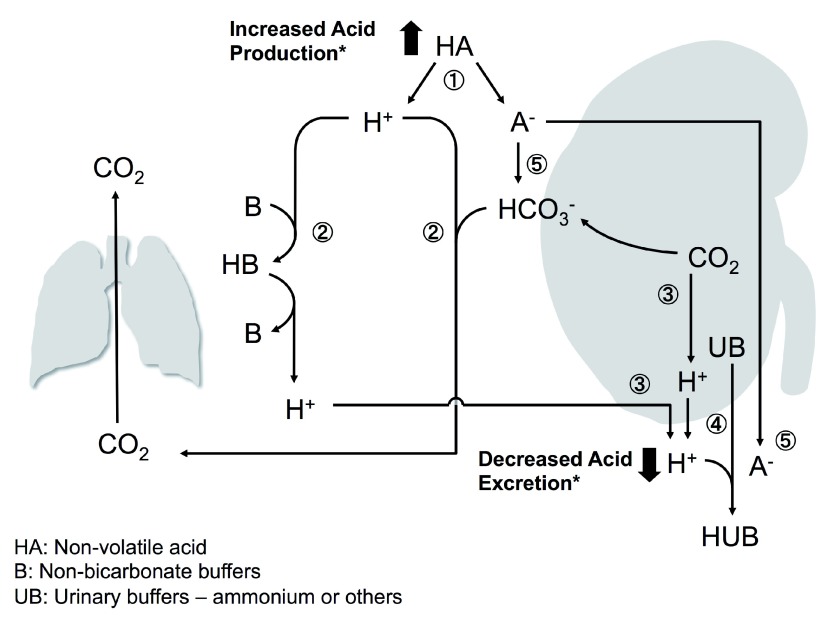
Excretion of acid and ways to jeopardize the system. 1. A strong non-volatile acid HA dissociates to release H ^+^ and poses an immediate threat to plasma pH. 2. Bicarbonate buffers the H ^+^ and generates CO _2_, which is expelled in the lungs and results in depletion of body HCO _3_^-^. Non-bicarbonate buffers (collectively referred to as B) carry the H ^+^ until the kidneys excrete it. 3. The kidneys split CO _2_ into H ^+^ and HCO _3_^-^ and selectively secrete H ^+^ into the lumen and HCO _3_^-^ into the blood. In addition, any excess H ^+^ from the body fluid is also excreted. 4. Most H ^+^ excreted in the urine is carried by urinary buffers (UBs). 5. Some organic anions (A) (e.g. lactate, ketoanions) can be metabolized to regenerate the HCO _3_^-^. If A is not metabolizable (e.g. phosphate or sulfate), it is excreted in the urine. * Two possible ways by which metabolic acidosis can occur.

Humans possess homeostatic mechanisms that maintain acid-base balance ( Figure 1). One utilizes both bicarbonate and non-bicarbonate buffers in both the intracellular and the extracellular milieu in the immediate defense against volatile (mainly CO _2_) and non-volatile (organic and inorganic) acids before excretion by the lungs and kidneys, respectively. Renal excretion of non-volatile acid is the definitive solution after temporary buffering. This is an intricate and highly efficient homeostatic system. Derangements in over-production, under-excretion, or both can potentially lead to accumulation of excess acid resulting in metabolic acidosis ( Figure 1).

**Figure 2.**
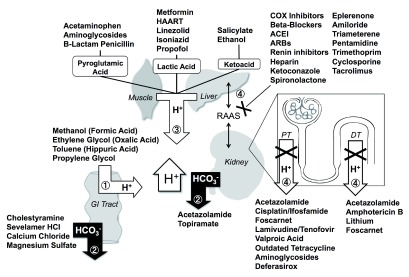
Mechanisms of drug-induced metabolic acidosis. 1. Increased exogenous ingestion of acidic precursors that are converted into strong acids. 2. Loss of alkali from kidney or GI tract. 3. Increased endogenous production of strong organic acids. 4. Compromised renal net acid excretion by inhibition of the renin-angiotensin-aldosterone system (RAAS), impaired proximal tubule (PT), or distal tubule (DT) H ^+^ secretion.

Drug-induced metabolic acidosis is often mild, but in rare cases it can be severe or even fatal. Not only should physicians be keenly aware of this potential iatrogenic complication but they should also be fully engaged in understanding the pathophysiological mechanisms. Metabolic acidosis resulting from drugs and/or ingestion of toxic chemicals can be grouped into four general categories ( Figure 2):

1. Drugs as exogenous acid loads
2. Drugs leading to loss of bicarbonate in the gastrointestinal (GI) tract or kidney
3. Drugs causing increased endogenous acid production
4. Drugs that decrease renal acid excretion

Some medications cannot be placed into one single category, as they possess multiple mechanisms that can cause metabolic acidosis.

In suspected drug-induced metabolic acidosis, clinicians should establish the *biochemical diagnosis* of metabolic acidosis along with the evaluation of respiratory compensation and whether there is presence of mixed acid-based disorders ^2^, then convert the biochemical diagnosis into a *clinical diagnosis* with identification of the invading acid/drug ^3^. Next is to review the list of medications by history and record to determine whether any of the drugs are culprits in either the generation or the exacerbation of the disorder. Note that just because a patient has, for example, lactic acidosis and is on a drug that can potentially cause lactic acidosis does not mean that the two are causally related. Finally, if a drug is indeed causing some degree of metabolic acidosis, the clinician should make an appraisal of the benefits from the drug weighed against the severity of the metabolic complication to determine whether cessation of therapy is justified. For example, if a patient with problematic seizures is effectively controlled by topiramate, a mild degree of metabolic acidosis can be more tolerable than seizures.

## Drugs resulting in exogenous acid precursors

### Non-pharmaceutical agents: toxic alcohols, phenols, and ammonium chloride

Methanol ^4^, ethylene glycol ^5^, diethylene glycol ^6^, and isopropanol ^7^ are volatile alcohols that produce a high plasma osmolar gap (the alcohol itself and the aldehyde metabolite), pure high anion gap metabolic acidosis from their metabolism into strong carboxylic acids such as formic acid (from methanol), and a combination of oxalic, glyoxylic, and glycolic acid (from ethylene/diethylene glycol). Isopropanol alcohol, due to the absence of an alpha-carbon, could only be metabolized to a keto- group and contributes to an osmolar gap but not high anion gap metabolic acidosis in poisoning encounters. Toluene abuse with glue or paint thinner sniffing can cause hippuric metabolic acidosis that presents with a normal plasma anion gap but elevated urinary osmolar gap because of the rapid clearance of hippurate ^8^. Note that the time at which blood is sampled may reveal variable osmolar and anion gap. When the hydroxyl group is metabolized to carboxyl with a low pKa, there will not be an osmolar gap due to the contemporaneous consumption of bicarbonate; however, the metabolite between hydroxyl and carboxyl is an aldehyde, which still contributes to an osmolar gap but not an anion gap.

Ammonium chloride is not usually abused but is used extensively by investigators to study overproduction acidosis and used outside the laboratory ^9^. There is a rise in acid excretion and a fall in serum HCO _3_^-^ concentration that remains constant after initial drop ^10, 11^.

### Overproduction acidosis from pharmaceutical agents

The excessive use of amino acids with a net positive charge would result in liberation of H ^+^ during metabolism (arginine and lysine) in parenteral alimentation with inadequate concomitant administration of alkali ^12^. Another example in this category is propylene glycol (1,2-propanediol [PG]), a common hygroscopic and emulsifying agent that is metabolized to lactate ^13^. The U.S. Food and Drug Administration classified PG as GRAS (generally recognized as safe). The recommended maximum daily intake of PG should be less than 25 mg/kg/day (equivalent to 21 mmol/day for a 70 kg person) ^14^. Each drug injection may have very different amounts of PG. Clinically significant toxicity is seen only in rapid, massive, and protracted parenteral administration of high quantities, especially in patients with renal impairment. PG intoxication from intravenous vitamin therapy was reported in pediatric patients who developed stupor ^15^. Intoxication with lactic acidosis and hyperosmolality were found during treatment of schizophrenia ^16^, with the use of intravenous benzodiazepines ^13, 17^, etomidate ^18^, nitroglycerin ^19^, and barbiturates ^20^, all with PG as a vehicle. Approximately 55% of PG undergoes oxidation to propionaldehyde and pyruvic, acetic, and lactic acid, while the remainder is excreted unchanged in the urine ^21, 22^. Some studies have demonstrated PG-injured proximal tubular cells, leading to impaired renal acidification ^20, 23^. Patients with hepatic dysfunction, renal insufficiency, and diabetic ketoacidosis are more susceptible to PG toxicity and development of lactic acidosis ^24^.

## Drugs causing external base loss

### Renal loss of bicarbonate

Carbonic anhydrases (CAs) are critical enzymes for bicarbonate reabsorption. Acetazolamide is a commonly used CA inhibitor in the treatment of ocular and convulsive disorders. It causes bicarbonaturia and a mild degree of hyperchloremic metabolic acidosis ^25^. There have also been reports of symptomatic anion gap metabolic acidosis associated with acetazolamide therapy in elderly patients ^26^ and in those with impaired renal function ^26, 27^ and diabetes mellitus ^28^. Severe metabolic acidosis may result from inhibition of pyruvate carboxylase and mitochondrial damage ^29^. Ocular solution of CA inhibitor-induced acidosis is rare but has been reported ^30^.

Topiramate is approved for the treatment of seizure, as a migraine headache prophylaxis, and for weight loss, with off-label use for bipolar disorder, obesity, neuropathic pain, and smoking cessation ^31^. It inhibits CA II, IV, and XII ^31^. Topiramate generates a mild hyperchloremic metabolic acidosis ^32, 33^ but increases urinary pH and drastically lowers urinary citrate excretion, thus increasing the risk for calcium phosphate urolithiasis ^34, 35^.

The sulfonamide class of drugs also has CA inhibitory activity. Topical application and absorption over large areas in burn patients can cause extremely high blood levels and systemic CA inhibition ^36^. This results in more than mere renal bicarbonate loss but rather a systemic disequilibrium syndrome.

### Gastrointestinal loss of bicarbonate

Cholestyramine is an oral agent for treating hypertriglyceridemia and cholestasis by binding and sequestering bile acids from the entero-hepatic circulation; the non-absorbable complexes are eventually excreted in the stool ^37^. In the GI tract, cholestyramine also binds phosphate, sulfate, and bicarbonate, leading to potential loss of bicarbonate from the body. Under normal conditions, this is easily corrected by renal regeneration of bicarbonate. However, patients with impaired renal function are at risk of hyperchloremic acidosis ^37– 39^.

Sevelamer hydrochloride is a non-reabsorbable phosphate binder. Dialysis patients on sevelamer hydrochloride have lower mean serum bicarbonate concentration during and at the end of therapy compared to those treated with calcium acetate ^40, 41^. The chloride released upon phosphate stimulates bicarbonate secretion by the gut via chloride-bicarbonate exchange ^40^. This secretion coupled with defective ability to regenerate bicarbonate in renal patients leads to hyperchloremic acidosis. This complication is avoided by using sevelamer carbonate, which binds phosphate and releases carbonate instead ^42^, or by bixalomer, which contains no chloride, and seems to demonstrate equal efficacy of phosphate binding with no evidence of acidosis in clinical trials ^43, 44^. Laxative abuse, calcium chloride, and magnesium sulfate could also cause hyperchloremic acidosis because the secreted bicarbonate from the pancreas is trapped by calcium and magnesium ^45– 47^ and then excreted in stools.

## Drugs causing increased endogenous acid production

### Lactic acidosis

**Figure 3.**
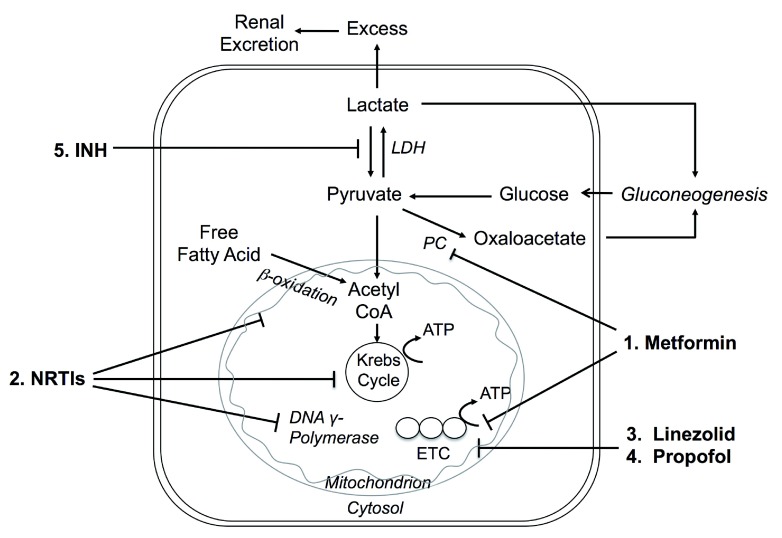
Mechanisms of drug-induced lactic acidosis. 1. Metformin inhibits pyruvate carboxylase (PC) → inhibits hepatic gluconeogenesis ^146^ → excess lactate ^84^. Metformin also inhibits complex I of the mitochondrial electron transport chain (ETC) ^84^ → increases NADH/NAD ^+^ ratio → blocks the entry of pyruvate into the tricarboxylic acid (TCA) cycle ^147^. LDH = lactate dehydrogenase 2. *In vitro*, nucleoside reverse transcriptase inhibitors (NRTIs) inhibit β-oxidation, the tricarboxylic acid (Krebs) cycle, and DNA γ-polymerase → mitochondrial dysfunction and loss of transcription of essential enzymes → hepatic steatosis (increased triglycerides), myopathy, pancreatitis, nephrotoxicity, and lactic acidosis ^68^. 3. Linezolid may cross-react with mammalian cellular processes → disrupts mitochondrial protein synthesis involved in ETC ^75, 148^. 4. Propofol may inhibit coenzyme Q and cytochrome C at Complex IV in ETC, and also inhibit mitochondrial fatty acid metabolism ^88^. 5. Isoniazid inhibits metabolism of lactate to pyruvate ^82^.

Lactic acid is produced under basal metabolic conditions and H ^+^ ions are released. Normally, an equivalent amount of H ^+^ ions is consumed when the liver and renal cortex utilize lactate for gluconeogenesis or oxidize it to water and CO _2_ so that acid-base balance remains undisturbed ( Figure 3). Lactic acidosis is arbitrarily classified into overproduction of lactate (type A), underutilization of lactate (type B), or both ^48^. Type A lactic acidosis is associated with generalized or regional tissue hypoxia, while type B is seen in patients with metabolic abnormalities in malignancy, hepatic dysfunction, diabetes mellitus, congenital enzymatic deficiency, and drugs or toxins ^45, 49^. In 1995, metformin replaced phenformin, a notorious inducer of lactic acidosis, and became the primary biguanide used today ^50^. Post marketing safety surveillance revealed no cases of fatal lactic acidosis ^51^. There are still reports of metformin-associated lactic acidosis (MALA) ^52, 53^ with proposed mechanisms shown in Figure 3.

Most cases of MALA were associated with some underlying conditions such as acute renal failure induced by volume depletion, other potential nephrotoxic agents and concurrent use of radio-contrast media, or hepatic insufficiency ^54– 58^. Blood pH and lactate levels are not prognostic in MALA ^59^. Although the incidence of MALA is low, once developed, the mortality can be staggeringly high ^52^, particularly in the critical care setting, so discontinuation is advised in a patient with impending renal and multi-organ failure. Recently, a less restrictive guideline is proposed on metformin usage in patients of stable chronic kidney disease ^60– 63^. In general, the mortality of MALA decreased from 50% to 25% from the 1960s to the present ^64^.

Highly active antiretroviral therapy (HAART) has led to dramatic reductions in HIV-associated morbidity and mortality ^65^ ( Figure 3). However, lactic acidosis complicated this therapy, especially with the nucleoside and nucleotide reverse transcriptase inhibitor (NRTI)-based regimens: didanosine, stavudine, lamivudine, zidovudine, and abacavir ^66– 71^. Combined use of these drugs further increases the risk of lactic acidosis ^72^. Moreover, didanosine ^73^, cidofovir ^74^, lamivudine, and stavudine ^75^ could cause Fanconi syndrome with pan-proximal tubular dysfunction leading to exacerbation of the acidosis and reduction of the plasma anion gap. The mortality of HAART-induced lactic acidosis can be as high as 50% ^76^.

Linezolid is a long-term antibiotic against serious resistant Gram-positive organisms ^77, 78^ with adverse effects including bone marrow toxicity, optic/peripheral neuropathy, and lactic acidosis ^77, 79, 80^. Concurrent use of selective serotonin uptake inhibitors such as citalopram and sertraline may predispose patients to lactic acidosis ^81, 82^. The vast majority of lactic acidosis occurred in the elderly and those receiving prolonged treatment, and most resolved upon cessation of linezolid ^80^. Children receiving linezolid appeared to suffer lactic acidosis earlier during treatment ^83^ ( Figure 3).

Isoniazid is commonly used to treat tuberculosis ^84^. Dosing more than 300 mg/day can lead to refractory grand mal or localized seizure, coma, and lactic acidosis ^84– 86^. Some suggested acidosis stems from excessive muscle activity during refractory seizure ^86, 87^, and slow reversal was observed in the postictal period. One proposed mechanism is inhibition of conversion of lactate to pyruvate ^84, 87– 89^ ( Figure 3).

Propofol is commonly used for induction and maintenance of anesthesia, sedation, and interventional procedures. Two cases were reported on propofol-associated severe metabolic acidosis ^90, 91^. Risk factors include severe head injury, critical illness, prolonged administration (>48 hours) of large doses (>4 mg/kg/hour, equivalent to 1.6 mmol/hour for a 70 kg person), and inborn errors of fatty acid oxidation ^90, 92^ ( Figure 3).

### Ketoacidosis

Ketosis develops when metabolism of fatty acid exceeds the removal of ketoacids (acetoacetic and β-hydroxybutyric). Typically there is insulin deficiency and/or resistance coupled with elevated glucagon and catecholamine. Glucose utilization is impaired and lipolysis is increased, augmenting the delivery of glycerol, alanine, and fatty acids for ketoacid generation ^45, 93^.

Overdose with salicylates in children commonly produces high anion gap acidosis, while adults exhibit a mixed respiratory alkalosis and metabolic acidosis. Metabolic acidosis occurs during salicylate toxicity due to uncoupling of oxidative phosphorylation and interfering with the Krebs cycle ^45, 86^, resulting in accumulation of lactic acid and ketoacids in as many as 40% of adult patients with salicylate poisoning ^45, 94, 95^. The anion gap is mainly composed of ketoanions and lactate, while salicylate anion seldom exceeds 3 mEq/L.

Alcoholic ketoacidosis occurs when ethanol is abused chronically in the setting of poor carbohydrate intake and volume contraction. Ketosis resolves when the ethanol intake is interrupted and the patient is provided with nutrients and fluid, which stimulates insulin secretion and promotes the regeneration of bicarbonate from the metabolism of ketoacid anions ^45^.

### Pyroglutamic acidosis

The γ-glutamyl cycle produces glutathione, which is involved in the inactivation of free radicals, detoxification of many compounds, and amino acid transport ^45, 96, 97^. Acetaminophen can deplete glutathione, leading to increased formation of γ-glutamyl cysteine, which is converted and accumulated as pyroglutamic acid (5-oxoproline) ^45, 97^. Patients at risk include those with malnutrition, sepsis, alcohol abuse, underlying liver disease, and/or renal insufficiency ^96^. Acetaminophen ingestion alone may not cause pyroglutamic acidosis, but synergistic interaction between acetaminophen and the other factors as noted above ^96^ can. Concomitant use of other drugs such as aminoglycoside and β-lactam penicillin is reported to increase the risk ^98^.

## Drugs causing decreased renal acid excretion

### Syndromes of hyper-hypokalemia and reduced distal hydrogen secretion

**Figure 4.**
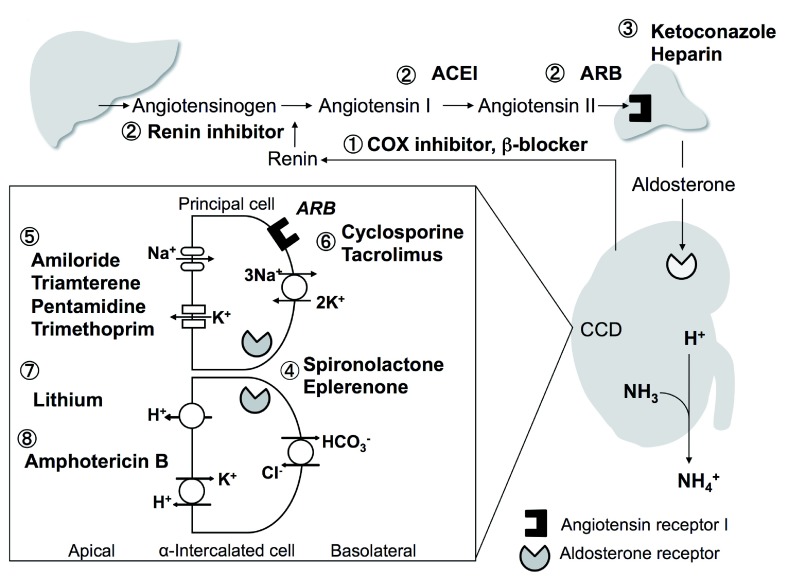
Mechanisms of drug-induced distal H ^+^ secretion. 1. Cyclooxygenase (COX) inhibitors and β-blockers interfere with release of renin, leading to hyperkalemia with metabolic acidosis ^43, 101^. 2. Angiotensin-converting enzyme inhibitors (ACEIs), aldosterone receptor blockers (ARBs), and renin inhibitors all interfere with the renin-angiotensin-aldosterone system (RAAS), causing hyperkalemia with hyperchloremic metabolic acidosis ^102– 104^. 3. Heparin ^105^ and ketoconazole ^106, 107^ interfere with aldosterone synthesis. 4. Spironolactone and eplerenone block aldosterone receptors ^43, 108^. 5. Na ^+^ channel blockers lead to reduced net negative charge in lumen in cortical collecting ducts (CCD), which reduces K ^+^ and H ^+^ excretion and causes hyperkalemia and acidosis ^43, 108– 111^. 6. Calcineurin inhibitors interfere with Na, K-ATPase in the principal cell decreasing transepithelial K secretion and H ^+^ secretion, cause vasoconstriction of afferent glomerular arterioles, and decrease glomerular filtration rate and alter filtration fraction ^112, 113^. 7. Lithium causes a voltage-dependent defect for H ^+^ secretion and decreases H ^+^-ATPase activity ^114– 116^. 8. Amphotericin B binds to sterol in mammalian cell membranes ^106, 107^ forming intramembranous pores which increase permeability and back diffusion of H ^+^.

Both angiotensin II and aldosterone are stimulators of the H ^+^-ATPase α-intercalated cells in the cortical collecting tubule ^99, 100^, adding H ^+^ into the urinary luminal. Inhibition of the renin-angiotensin-aldosterone system (RAAS), which leads to secondary inhibition of the H ^+^-ATPase, can lead to decreased H ^+^ secretion and metabolic acidosis. Additionally, inhibition of the RAAS reduces Na ^+^ reabsorption, which reduces the luminal electronegativity and reduces H ^+^ excretion by the H ^+^-ATPase ^100^. The same mechanisms can cause hyperkalemia, which can in turn reduce stimulation of the H ^+^/K ^+^-ATPase ^101^. Hyperkalemia suppresses ammoniagenesis in the proximal tubule, impairs NH _4_^+^ transport in the medullary thick ascending limb, and reduces medullary interstitial ammonium concentration, all of which can lower urine acid excretion ^45, 102^. Therefore, any drug that affects the RAAS or causes hyperkalemia can increase the risk of metabolic acidosis. These drugs include the following ( Figure 4):

- Cyclooxygenase (COX) inhibitors ^45, 103^
- β-adrenergic receptor blockers ^45, 104^
- Angiotensin-converting enzyme inhibitors (ACEIs), angiotensin II receptor blockers (ARBs), and direct renin inhibitors ^104– 106^
- Heparin ^107^ and ketoconazole ^108, 109^
- Spironolactone and eplerenone ^45, 110^
- Potassium-sparing diuretics: amiloride and triamterene ^45, 110^
- Pentamidine and trimethoprim ^111– 113^
- Calcineurin inhibitors: cyclosporine and tacrolimus ^114, 115^

When these drugs are administered in combination, there is increased risk for hyperkalemia and metabolic acidosis, especially in patients with diabetes, chronic kidney disease, and liver disease ^45^.

In contrast, patients with classic distal renal tubular acidosis (dRTA) generally have hypokalemic hyperchloremic metabolic acidosis. The metabolic acidosis results from the inability to acidify urine in the distal nephron and impaired excretion of NH4 ^+^^100^. Inherited forms of dRTA have defects in the basolateral HCO _3_^-^/Cl ^-^ exchanger, B1 or A4 subunits of the H ^+^-ATPase, or CA. Some medications can mimic these defects by altering membrane permeability and causing leaky pathways ^45^. Amphotericin B ^108, 109^, lithium ^116– 118^, and foscarnet ^119^ are known to cause leak and lead to hypokalemic hyperchloremic metabolic acidosis ( Figure 4).

### Drugs causing Fanconi syndrome and proximal renal tubular acidosis

**Figure 5.**
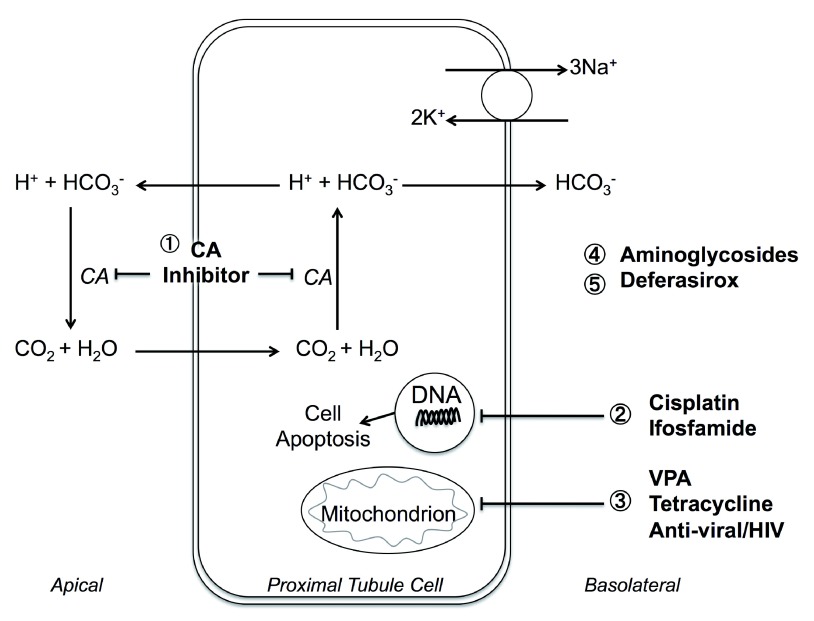
Mechanisms of proximal tubule (PT) and drug-induced Fanconi syndrome. 1. CA inhibitors ^25^ cause bicarbonaturia and hyperchloremic metabolic acidosis in the elderly ^26^ and patients with renal failure ^27^ and diabetes ^28^. 2. Antineoplastic platinum-containing agents ^126, 127^ and DNA-alkylating agents ^128– 130^ damage proximal tubule cells through accumulation and induced cell apoptosis. 3. Anti-viral/HIV drugs ^75, 121– 124^, valproic acid (VPA) ^131– 133^, and outdated tetracycline ^134– 136^ interfere with mitochondrial function within proximal tubule cells, leading to tubular dysfunction. 4. Aminoglycosides ^137, 148, 149^ induce acidosis with unclear mechanisms ^150^. 5. Deferasirox ^139– 143^ increases hemodynamic iron removal, causes vacuolization of proximal tubular epithelial cells ^142^, and elevates iron absorption in various organs. All could lead to acidosis.

The proximal tubule is the initial step in renal acidification and is essential in maintaining acid-base homeostasis by reclaiming 80% of filtered bicarbonate (HCO _3_^-^) ( Figure 5). Bicarbonate reabsorption occurs by luminal H ^+^ excretion and HCO _3_^-^ extrusion back into the blood at the basolateral membrane ^100^. CAs catalyze the reaction: CO _2_ + H _2_O → HCO _3_^-^+ H ^+^. If proximal HCO _3_^-^ reclamation is impaired, more HCO _3_^-^ is delivered to the distal tubule, which has limited capacity for HCO _3_^-^ reabsorption. Bicarbonaturia ensues and net acid excretion decreases, which eventually leads to metabolic acidosis ^45, 120^. Generalized proximal tubule dysfunction is termed Fanconi syndrome. Potential drugs that could induce Fanconi syndrome include the following ( Figure 5):

- CA inhibitors (e.g. acetazolamide) ^25^.
- Anti-viral/HIV drugs (e.g. lamivudine, stavudine ^75^ and tenofovir ^121– 124^). Most tenofovir-induced cases are subclinical ^125^
- Platinum-containing agents (e.g. cisplatin ^126, 127^) and DNA alkylating agents (e.g. ifosfamide ^128– 130^) are common proximal tubule toxins. Note that cyclophosphamide, structural isomer of ifosfamide, can also cause hemorrhagic cystitis but is not nephrotoxic by producing less chloroacetaldehyde ^128^
- Valproic acids (VPAs) ^131– 133^
- Outdated tetracycline ^134– 136^
- Aminoglycoside ^137^ accumulation in proximal tubule would lead to nephrotoxicity with an unclear mechanism; however, incidence decreased recently due to a better monitoring strategy ^138^
- Deferasirox ^139– 143^

Many other agents such as fumaric acid ^144^, suramin ^145^, and imatinib ^146^ have also been associated with Fanconi syndrome in case reports. This field remained to be further explored as proximal tubule toxicity is common due to the existence of multiple drug transporters at the surface membrane, leading to very high uptake of drugs by this segment ^147^.

## Conclusion

In summary, metabolic acidosis can occur as a side effect of therapy. Instead of memorizing the catalogue of drugs, clinicians should classify these agents based on their pathophysiologic mechanisms to facilitate the recognition of potential causal relationships. Some of these side effects are inferred from empirical observations, but some have undergone extensive studies to determine the pathogenesis of metabolic acidosis. We hope that this review will intrigue our readers to experience that eureka moment identifying unrecognized explanations for metabolic acidosis in patients or to partake in extending clinical observations to clinical investigations.

## Abbreviations

ACEI, Angiotensin-converting enzyme inhibitor; ARB, Aldosterone receptor blocker; CA, Carbonic anhydrase; COX, Cyclooxygenase; dRTA, Distal renal tubular acidosis; FDA, Food & Drug Administration; GRAS, Generally recognized as safe; GI, Gastrointestinal; HAART, Highly active antiretroviral therapy; MALA, Metformin-associated lactic acidosis; NAD, Nicotinamide Adenine Dinucleotide; PG, Propylene glycol; RAAS, Renin-angiotensin-aldosterone system; VPA, Valproic acid.
